# Adolescent screen time, anxiety/depression, and alcohol/e-cigarette use: evidence from the ABCD study

**DOI:** 10.1186/s12889-025-25956-3

**Published:** 2025-12-17

**Authors:** Maria A. Parker, Moriah R. Harton, Paola P. Mattey-Mora, Joanna M. Streck

**Affiliations:** 1 Indiana University Bloomington, School of Public Health, Department of Epidemiology and Biostatistics, Bloomington, IN USA; 2 Department of Psychiatry, Indiana University School of Medicine, Indianapolis, IN USA; 3 Tobacco Research and Treatment Center, Division of General Internal Medicine Department of Medicine, Massachusetts General Hospital and Harvard Medical School, Boston, MA USA; 4 Department of Psychiatry, Massachusetts General Hospital and Harvard Medical School, Boston, MA USA

**Keywords:** Adolescents, Anxiety, Depression, Screen time, Social media, Group-based trajectory modeling

## Abstract

**Background:**

Adolescents are spending more time on screens, which has been associated with anxiety, depression, and increased substance use, especially through social media. This study aimed to examine the prospective relationship between screen time and anxiety/depression and the moderating effect of alcohol/e-cigarette use.

**Methods:**

Data were drawn from the Adolescent Brain and Cognitive Development (ABCD) study, with participants aged 9–10 at baseline (*n* = 9,474). This cohort study analyzed longitudinal data collected between 2016 and 2022. Parents completed the Child Behavior Checklist (CBCL), which assessed anxiety/depression at the year 3 follow-up. Group-based trajectory models identified screen time/social media patterns, and multivariable linear models examined anxiety/depression, including an interaction term for alcohol/e-cigarette use.

**Results:**

Four groups of screen time were identified. Higher screen time groups showed higher anxiety and depression (*p* ≤ 0.001) compared to the persistent low screen time group. Five groups of social media use were identified. The gradual increase to moderate time on social media group showed lower anxiety (*p* = 0.031) but similar depression t-scores compared to the largest group with persistent low use of social media. In contrast, the other three groups with high time spent on social media by the end of the study had similar anxiety but higher depression t-scores relative to the largest group (*p* ≤ 0.014). Alcohol/e-cigarette use did not moderate these relationships.

**Discussion:**

Higher screen time was associated with higher anxiety and depression, while moderate social media use was associated with lower anxiety. High social media use was associated with higher depression. These findings highlight nuanced mental health impacts of screen behaviors as well as the need for public health strategies that emphasize moderation and promote balanced screen behaviors.

**Supplementary Information:**

The online version contains supplementary material available at 10.1186/s12889-025-25956-3.

## Background

Adolescence is a developmental stage marked by social, cognitive, and biological changes that coincide with the onset of mental health conditions, raising important public health considerations [1]. Nearly half of lifetime diagnoses of mental health disorders occur by the mid-teens and three-fourths by the mid‐20s [2]. This phase is marked by hormonal and brain changes as well as social transitions [3], all of which can contribute to the onset of anxiety and depression. Additionally, adolescence is when substance use initiation commonly occurs [4, 5], coinciding with peak levels of screen time.

Screen time, defined as time spent on electronic media (e.g., tablets, smartphones, computer), has become an integral part of adolescents’ daily life, encompassing education, entertainment, and socialization. Screen time encompasses multiple activities and purposes, including information seeking and learning, which may differentially associate with mental health outcomes [6]. In the United States, 90–95% of adolescents have access to a smartphone or a computer and engage in activities such as video streaming, gaming, texting, and social media use [7]. As adolescents age, overall screen time increases, with notable shifts toward greater social media engagement [7].

The causal relationship between screen time and mental health is under debate, with indeterminate underlying biological mechanisms. Screen time can increase sedentary behavior, reducing mental health through physical activity’s interactive relationship with screen time [8, 9]. Indirect pathways have been suggested, such as associations between screen use and internalizing problems via changes in relationships or social solitude [9] and those observed via sleep problems, unhealthy eating, body weight issues, and victimization [8], though evidence is mixed.

Recent studies have examined the association between screen time and mental health, especially anxiety and depression, which has become an evolving area of scientific study. While some studies suggest links between screen time and poor mental health [10–16], other work indicates minimal or even positive associations [17–19]. Meta-analyses of reductions in social media show no significant mental health improvements [20].

One scoping review highlighted that increased screen time was related to decreased self-esteem, greater mental health issues, slowed learning and acquisition, and an increased risk of premature cognitive decline [21]. Another study found social media use was linked to higher online harassment, sleep issues, low self-esteem, and negative body image, all of which were associated with increased depression scores [11]. However, longitudinal evidence on the relationship between screen time and anxiety is inconsistent and varies by device type and usage patterns, with overall effect sizes remaining small [22].

The relationship between screen time and substance use is an emerging concern. Screen time, particularly through screen-based activities, may increase the risk of substance use in adolescents [23], though quality and consistency of findings vary. Given that peak screen time use overlaps with typical age of substance use initiation [4, 5] and that adolescents who use substances often have higher anxiety and depression [24, 25], substance use may modify the association between screen time and anxiety/depression in adolescence. However, this interaction remains largely unexplored.

For youth and young adults receiving mental health screening and intervention, high screen time has been associated with increased risk of substance use (i.e., nicotine, alcohol, cannabis), depression, and anxiety [24]. Anhedonia, or a reduced ability to feel pleasure, has been found to mediate the relationship between screen time and substance use (i.e., alcohol, cigarettes) [26]. Furthermore, substance use may interact with screen time through shared neurobiological reward and coping mechanisms that modulate stress regulation, suggesting a potential moderating effect on anxiety and depression. During adolescence, a developmental asynchrony emerges between the mesolimbic system—responsible for reward sensitivity, novelty seeking, and risk-taking—and the prefrontal cognitive control system, which governs impulse regulation and executive functioning [27]. This imbalance has been associated with the initiation and escalation of addictive-like behaviors, including substance use [28] and excessive screen or social media engagement [29], as well as heightened emotional reactivity that increases susceptibility to anxiety and depression [30, 31]. Since most studies have not accounted for substance use [24, 26], further investigation is needed.

Unlike most prior studies, which were cross-sectional or focused only on screen time, this study uses longitudinal data to prospectively examine how trajectories of screen time and social media use relate to later anxiety and depression and tests whether substance use moderates this association. Using a large, demographically diverse sample of adolescents, we explored how different patterns of screen time and social media use are associated with symptoms of anxiety and depression, as well as the moderating role of alcohol and electronic cigarettes as markers of substance use (other substances assessed, cigarette smoking and cannabis use, had zero prevalence). Understanding this complex longitudinal relationship is crucial for informing public health initiatives and developing interventions and policies to support adolescent well-being in an increasingly digital world. Given the ubiquity of screen time, even small effects might translate into meaningful public health burdens when considered at the population level.

## Methods

### Study design and participants

Data came from the Adolescent Brain Cognitive Development (ABCD) Study, a longitudinal study of United States (US) children [32]. The ABCD study is a large, nationally representative cohort, providing population-level insights relevant to adolescent health policy and prevention strategies. This cohort began in 2016 with 11,880 participants aged 9–10 across 21 research sites. Annual follow-ups include assessments of behavior, health, and environment. The current study used data up to the year 3 follow-up (age 12–13; ABCD Data Release 5.1; January 2022). We analyzed 9,474 participants with screen time data for at least one of the four time points, alcohol and e-cigarette data, and anxiety/depression data at the year 3 follow-up. This study is reported following the Strengthening the Reporting of Observational Studies in Epidemiology (STROBE) reporting guideline. Our data analysis of de-identified data was deemed not human subjects research by Indiana University – Bloomington’s Institutional Review Board (#16479).

### Measures

### Screen time (primary exposure) and social media (secondary exposure)

Screen time was self-reported as average hours spent on electronic devices (e.g., TV, texting, gaming, social media), aggregated into a weekly measure (5*weekday + 2*weekend) [33–35]. Social media was self-reported separately in hours per weekday/weekend and standardized across follow-ups to ensure comparability with the general screen time variable. Screen time captured total electronic media exposure across modalities including time on social media, whereas social media use was examined separately to reflect interactive, peer-oriented online behaviors.

### Mental health outcomes

Anxiety and depression were assessed using the Child Behavior Checklist (CBCL) Diagnostic and Statistical Manual (DSM)-5 Scale, completed by parents at follow-up three. ABCD study t-scores were used, with higher scores indicating greater symptom severity (range: 50–97). For anxiety/depression, the CBCL has good convergence with clinical diagnosis [36].

### Substance use as a moderator

Substance use was measured as alcohol (full drink in past six months) and e-cigarette use (two or more puffs in past six months). Due to low prevalence (0.8% and 0.9%, respectively), these were combined into a single binary variable (yes/no). Because we aimed to examine non-experimental substance use as a moderator, we intended to also include cigarette smoking and cannabis use, but they both had zero prevalence in our sample. Use of alcohol and e-cigarette is the most common within this age group [37, 38]. Previous studies on adolescent screen time have operationalized substance use as *trying* alcohol and trying cigarettes [26], and nicotine, alcohol, and cannabis use in individuals in a screening, brief intervention and referral to treatment (SBIRT) program [24].

### Covariates

Baseline covariates included age in years, sex (male, female), race/ethnicity (White, Black, Hispanic, Asian, other), annual household income (<$50,000, $50,000-$99,999, $100,000+, unknown), and parental education (less than high school, high school graduate, some college, college graduate, more than a Bachelor’s degree, unknown).

### Statistical analysis

Group-based trajectory modeling (GBTM) identified screen time and social media usage patterns over time. Models with two to six groups were tested, selecting the best fit based on Bayesian Information Criterion (BIC), polynomial order, entropy (> 0.8), posterior probability (> 0.7), classification, and group size (> 2.5% of the sample) [39, 40]. Model fit indices for 2–6 class solutions are provided in Supplementary Tables 1 to ensure transparency in trajectory selection. Multiple linear regression models examined the association between screen time trajectories and anxiety/depression t-scores, adjusting for covariates. Substance use (i.e., alcohol/e-cigarette use) was included as a moderator via an interaction term with group membership. Exploratory analysis examined the association between time spent on social media and anxiety/depression due to its prolific use by adolescents [7]. Analyses were performed using Stata 17 (College Station, TX: StataCorp LLC) for GBTM and R 4.3.3 for regression modeling. Complex survey weights were applied to adjust for sampling design and attrition.

## Results

### Trajectory identification

**Fig. 1:**
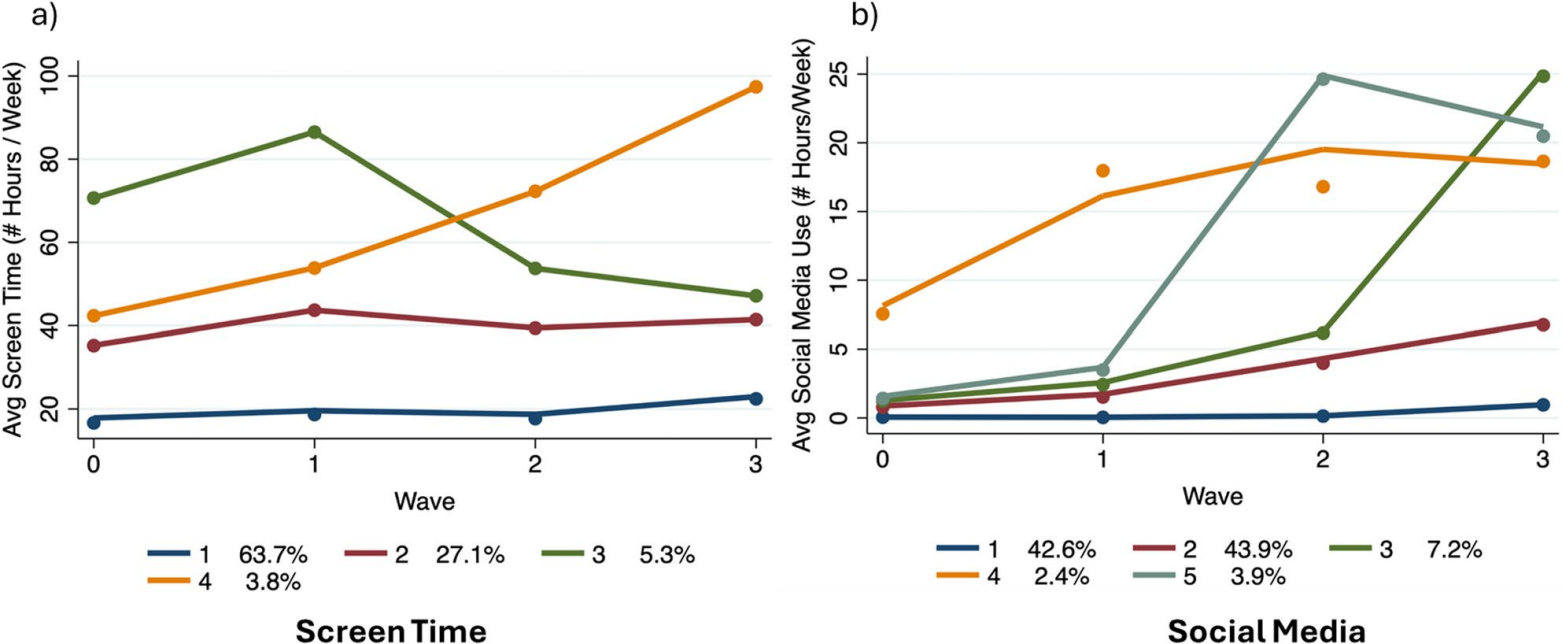
Group trajectories for screen time (**a**) and social media (**b**)

Four distinct screen time trajectory groups were identified: (1) Persistent Low Use (63.7%) – approximately 20 h/week of screen time (*n* = 6,130; order 3), (2) Consistent Moderate Use (27.1%) – around 40 h/week (*n* = 2,506; order 3), (3) Increasing-then-Decreasing Use (5.3%) – peaked early but stabilized at 40 h/week (*n* = 489; order 3), (4) Increasing Use (3.8%) – reached 100 h/week by the study’s end (*n* = 349; order 2) (Fig. 1a).

Additionally, five social media use trajectory groups were identified: (1) Persistent Low Use (42.6%) – less than 1 h/week (*n* = 4,255; order 2), (2) Gradual Increase to Moderate Use (43.9%) – reached ~ 7 h/week (*n* = 3,970; order 3), (3) Sharp Increase to High Use (7.2%) – peaked at 25 h/week (*n* = 678; order 3), (4) Moderate Increase to High Use (2.4%) – peaked at 19 h/week (*n* = 266; order 2), and (5) Fluctuating High Use (3.9%) – reached 20 h/week (*n* = 345, order 3) (Fig. 1b).

### Study sample characteristics and demographic differences

The overall study sample was 52% male. Race/ethnicity, household income, and parental education varied between groups for both screen time and social media. Alcohol and e-cigarette use was relatively consistent between groups (Tables 1 and 2).

**Table 1:** Background characteristics of the study sample, stratified by screen time trajectory group. Data from the ABCD study (2016–2022)

	Overall *n* = 9,474	Group 1 (Persistent Low Use) *n* = 6,130	Group 2 (Consistent Moderate Use) *n* = 2,506	Group 3 (Increasing-then-Decreasing Use) *n* = 489	Group 4 (Increasing Use) *n* = 349
Male	4,946 (52%)	3,012 (49%)	1,471 (59%)	278 (57%)	185 (53%)
Median Age at Baseline	9.92 (9.33, 10.42)	9.92 (9.33, 10.42)	9.92 (9.42, 10.50)	10.08 (9.42, 10.58)	10.00 (9.50, 10.50)
Race/Ethnicity					
Non-Hispanic White	5,233 (55%)	3,936 (64%)	1,068 (43%)	134 (27%)	95 (27%)
Non-Hispanic Black	1,163 (12%)	376 (6.1%)	477 (19%)	187 (38%)	123 (35%)
Hispanic	1,886 (20%)	1,056 (17%)	640 (26%)	106 (22%)	84 (24%)
Non-Hispanic Asian	203 (2.1%)	179 (2.9%)	19 (0.8%)	2 (0.4%)	3 (0.9%)
Non-Hispanic Other	989 (10%)	583 (9.5%)	302 (12%)	60 (12%)	44 (13%)
Annual Household Income					
<$50K	2,283 (24%)	1,037 (17%)	835 (33%)	237 (48%)	174 (50%)
$50K - <$99K	2,562 (27%)	1,607 (26%)	747 (30%)	114 (23%)	94 (27%)
$100K +	3,936 (42%)	3,104 (51%)	705 (28%)	87 (18%)	40 (11%)
Unknown	693 (7.3%)	382 (6.2%)	219 (8.7%)	51 (10%)	41 (12%)
Highest Parental Education					
< High school	494 (5.2%)	240 (3.9%)	184 (7.3%)	36 (7.4%)	36 (7.4%)
High school	876 (9.2%)	395 (6.4%)	329 (13%)	90 (18%)	62 (18%)
Some College	2,661 (28%)	1,311 (21%)	962 (38%)	226 (46%)	162 (46%)
College	2,851 (30%)	2,102 (34%)	602 (24%)	84 (17%)	63 (18%)
> Bachelor’s Degree	2,581 (27%)	2,077 (34%)	425 (17%)	52 (11%)	27 (7.7%)
Unknown	11 (0.1%)	5 (< 0.1%)	4 (0.2%)	1 (0.2%)	1 (0.3%)
Alcohol/E-cigarette use					
No	9,299 (98%)	6,053 (99%)	2,440 (97%)	472 (97%)	334 (96%)
Yes	141 (1.5%)	61 (1.0%)	55 (2.2%)	11 (2.2%)	14 (4.0%)
Unknown	34 (0.4%)	16 (0.03%)	11 (0.4%)	6 (1.2%)	1 (0.3%)

**Table 2:** Background characteristics of the study sample, stratified by social media trajectory group. Data from the ABCD study (2016–2022)

	Overall *n* = 9,474	Group 1 (Persistent Low Use) *n* = 4,255	Group 2 (Gradual Increase to Moderate Use) *n* = 3,970	Group 3 (Sharp Increase to High Use) *n* = 678	Group 4 (Moderate Increase to High Use) *n* = 226	Group 5 (Fluctuating High Use) *n* = 345
Male	4,946 (52%)	2,620 (62%)	1,919 (48%)	241 (36%)	67 (30%)	99 (29%)
Median Age at Baseline	9.92 (9.33, 10.42)	9.75 (9.25, 10.29)	10.00 (9.42, 10.58)	10.00 (9.42, 10.50)	10.33 (9.75, 10.75)	10.00 (9.50, 10.50)
Race/Ethnicity						
Non-Hispanic White	5,233 (55%)	2,800 (66%)	2,014 (51%)	235 (35%)	76 (34%)	108 (31%)
Non-Hispanic Black	1,163 (12%)	249 (5.9%)	561 (14%)	160 (24%)	87 (38%)	106 (31%)
Hispanic	1,886 (20%)	664 (16%)	900 (23%)	195 (29%)	33 (15%)	94 (27%)
Non-Hispanic Asian	203 (2.1%)	123 (2.9%)	71 (1.8%)	4 (0.6%)	2 (0.9%)	3 (0.9%)
Non-Hispanic Other	989 (10%)	419 (9.8%)	424 (11%)	84 (12%)	28 (12%)	34 (9.9%)
Annual Household Income						
<$50K	2,283 (24%)	703 (17%)	1,068 (27%)	269 (40%)	100 (44%)	143 (41%)
$50K - <$99K	2,562 (27%)	1,223 (29%)	1,032 (26%)	154 (23%)	57 (25%)	96 (28%)
$100K +	3,936 (42%)	2,113 (50%)	1,524 (38%)	174 (26%)	44 (19%)	81 (23%)
Unknown	693 (7.3%)	216 (5.1%)	346 (8.7%)	81 (12%)	25 (11%)	25 (7.2%)
Highest Parental Education						
< High school	494 (5.2%)	114 (2.7%)	260 (6.5%)	62 (9.1%)	17 (7.5%)	41 (12%)
High school	876 (9.2%)	241 (5.7%)	436 (11%)	102 (15%)	43 (19%)	54 (16%)
Some College	2,661 (28%)	1,000 (24%)	1,197 (30%)	251 (37%)	95 (42%)	118 (34%)
College	2,851 (30%)	1,509 (35%)	1,077 (27%)	145 (21%)	38 (17%)	82 (24%)
> Bachelor’s Degree	2,581 (27%)	1,386 (33%)	996 (25%)	117 (17%)	32 (14%)	50 (14%)
Unknown	11 (0.1%)	5 (0.1%)	4 (0.1%)	1 (0.1%)	1 (0.4%)	0 (0%)
Alcohol/E-cigarette use						
No	9,299 (98%)	4,209 (99%)	3,897 (98%)	660 (97%)	210 (93%)	323 (94%)
Yes	141 (1.5%)	35 (0.8%)	63 (1.6%)	15 (2.2%)	10 (4.4%)	18 (5.2%)
Unknown	34 (0.4%)	11(0.3%)	10 (0.3%)	3 (0.4%)	6 (2.7%)	4 (1.2%)

Females had higher depression scores than males (β = 0.28, 95% CI: 0.04, 0.53), but no significant difference was seen in anxiety in terms of screen time. No statistically significant differences were found between females and males, for both anxiety and depression in terms of social media. Black and Asian participants had lower anxiety and depression scores than White participants, while Hispanic participants had slightly lower depression scores in both the screen time and social media models. Finally, higher household income was associated with lower anxiety and depression scores, whereas higher parental education was associated with higher anxiety and depression scores in both models (Table 3 and Table 4).

**Table 3:** Linear regression coefficients of screen time (hours/week) stratified by anxiety/depression, *n* = 9474

	Anxiety			Depression		
	Beta	95% CI	*p*-value	Beta	95% CI	*p*-value
Trajectory Group						
1	----	----	----	----	----	----
2	0.55	0.25, 0.85	**< 0.001**	1.22	0.92, 1.52	**< 0.001**
3	0.96	0.37, 1.55	**0.001**	1.64	1.05, 2.23	**< 0.001**
4	1.44	0.75, 2.12	**< 0.001**	2.94	2.36, 3.63	**< 0.001**
Substance Use						
No	----	----	----	----	----	----
Yes	0.39	-1.14, 1.92	0.617	1.30	-0.22, 2.83	0.093
Unknown	0.25	-2.72, 3.21	0.870	-1.21	-4.16, 1.82	0.424
Sex						
Male	----	----	----	----	----	----
Female	0.20	-0.04, 0.45	0.106	0.28	0.04, 0.53	**0.024**
Median Age at Baseline	-0.08	-0.28, 0.11	0.405	-0.11	-0.30, 0.09	0.287
Race/Ethnicity						
Non-Hispanic White	----	----	----	----	----	----
Non-Hispanic Black	-1.93	-2.36, -1.50	**< 0.001**	-2.23	-2.68, -1.82	**< 0.001**
Hispanic	-0.28	-0.63, 0.07	0.120	-0.61	-0.96, -0.25	**< 0.001**
Non-Hispanic Asian	-1.71	-2.66, -0.86	**< 0.001**	-1.98	-2.83, -1.12	**< 0.001**
Non-Hispanic Other	0.15	-0.27, 0.57	0.477	0.24	-0.17, 0.66	0.248
Annual Household Income						
<$50K	----	----	----	----	----	----
$50K - <$99K	-0.63	-1.00, -0.25	**0.001**	-0.58	-0.95, -0.20	**0.003**
$100K +	-1.12	-1.51, -0.72	**< 0.001**	-1.18	-1.51, -0.72	**< 0.001**
Unknown	-0.60	-1.12, -0.08	**0.023**	-0.46	-0.98, 0.05	**0.079**
Highest Parental Education						
< High school	----	----	----	----	----	----
High school	0.18	-0.50, 0.86	0.598	0.17	-0.50, 0.85	0.618
Some College	0.86	0.25, 1.47	0.005	0.86	0.25, 1.46	0.005
College	0.95	0.30, 1.59	**0.004**	0.81	0.16, 1.45	**0.014**
> Bachelor’s Degree	1.18	0.51, 1.84	**< 0.001**	1.07	0.41, 1.73	**0.001**
Unknown	0.06	-3.56, 3.67	0.975	-0.19	-3.79, 3.42	0.919
Trajectory Group *Substance Use						
2*Yes	0.89	-1.33, 3.11	0.431	1.41	-0.80, 3.63	0.211
3*Yes	2.16	-1.77, 6.08	0.282	9.29	5.38, 13.21	**< 0.001**
4*Yes	2.00	-1.56, 5.57	0.274	3.22	-0.34, 6.79	0.076
2*Unknown	2.20	-2.45, 6.85	0.354	1.57	-3.07, 6.21	0.507
3*Unknown	1.44	-4.26, 7.14	0.620	4.33	-1.36, 10.02	0.136
4*Unknown	-3.16	-15.41, 9.08	0.613	-1.99	-14.20, 10.21	0.749

*β *standardized regression coefficient, *CI*  confidence interval

### Trajectories and mental health outcomes

Compared to Group 1, the Persistent Low Use group, all other groups in the screen time trajectories had significantly higher anxiety and depression t-scores. The largest effect was seen in the Increasing Use group, which showed the greatest increase in both anxiety (β = 1.44, 95% CI: 0.75, 2.12) and depression (β = 2.94, 95% CI: 2.36, 3.63) (Table 3).

Regarding social media, higher social media use was associated with higher depression but not significantly with anxiety. The highest-use groups (Groups 3, 4, and 5) showed significantly higher depression t-scores compared to the Persistent Low Use group. However, the Gradual Increase to Moderate Use group showed lower anxiety t-scores than the low-use group (β = -0.30, 95% CI: -0.58, -0.03) (Table 4).

**Table 4:** Linear regression coefficients of social media use (hours/week) stratified by anxiety/depression, *n* = 9,474

	Anxiety			Depression		
	Beta	95% CI	*p*-value	Beta	95% CI	*p*-value
Trajectory Group						
1	----	----	----	----	----	----
2	-0.30	-0.58, -0.03	**0.031**	0.06	-0.22, 0.33	0.678
3	0.21	-0.31, 0.72	0.433	1.03	0.52, 1.55	**< 0.001**
4	0.52	-0.34, 1.38	0.240	1.08	0.22, 1.95	**0.014**
5	0.38	-0.32, 1.09	0.290	1.49	0.78, 2.19	**< 0.001**
Substance Use						
No	----	----	----	----	----	----
Yes	0.35	-1.66, 2.37	0.731	2.61	0.59, 4.63	**0.011**
Unknown	1.44	-2.14, 5.02	0.429	-0.88	-4.47, 2.71	0.630
Sex						
Male	----	----	----	----	----	----
Female	0.13	-0.12, 0.38	0.315	0.04	-0.21, 0.29	0.775
Median Age at Baseline	-0.04	-0.24, 0.16	0.691	-0.08	-0.28, 0.11	0.404
Race/Ethnicity						
Non-Hispanic White	----	----	----	----	----	----
Non-Hispanic Black	-1.70	-2.13, -1.27	**< 0.001**	-1.90	-2.33, -1.47	**< 0.001**
Hispanic	-0.21	-0.56, 0.14	0.248	-0.53	-0.89, -0.18	**0.003**
Non-Hispanic Asian	-1.76	-2.61, -0.90	**< 0.001**	-2.03	-2.99, -1.18	**< 0.001**
Non-Hispanic Other	0.25	-0.16, 0.67	0.237	0.41	-0.01, 0.82	0.057
Annual Household Income						
<$50K	----	----	----	----	----	----
$50K - <$99K	-0.67	-1.05, -0.29	**< 0.001**	-0.64	-1.01, -0.26	**< 0.001**
$100K +	-1.21	-1.60, -0.81	**< 0.001**	-1.29	-1.68, -0.89	**< 0.001**
Unknown	-0.60	-1.12, -0.08	**0.023**	-0.51	-1.03, 0.01	0.054
Highest Parental Education						
< High school	----	----	----	----	----	----
High school	0.22	-0.46, 0.90	0.521	0.26	-0.42, 0.94	0.461
Some College	0.91	0.30, 1.52	**0.003**	0.99	0.38, 1.60	**0.001**
College	0.88	0.23, 1.53	**0.008**	0.76	0.11, 1.40	**0.022**
> Bachelor’s Degree	1.07	0.41, 1.73	**0.002**	0.95	0.29, 1.62	**0.005**
Unknown	0.03	-3.59, 3.64	0.989	-0.08	-3.70, 3.55	0.967
Trajectory Group *Substance Use						
2*Yes	0.69	-1.83, 3.20	0.593	0.07	-2.45, 2.59	0.958
3*Yes	1.88	-1.82, 5.57	0.320	0.72	-2.99, 4.42	0.704
4*Yes	4.09	-0.24, 8.42	0.064	3.09	-1.26, 7.43	0.164
5*Yes	0.62	-2.89, 4.12	0.731	0.98	-2.45, 4.59	0.586
2*Unknown	-3.49	-8.68, 1.69	0.187	-1.01	-6.21, 4.19	0.704
3*Unknown	7.77	0.03, 15.51	0.049	9.84	2.08, 17.61	**0.013**
4*Unknown	-2.19	-8.27, 3.89	0.481	0.18	-5.92, 6.27	0.954
5*Unknown	3.96	-4.00, 9.92	0.405	1.39	-5.58, 8.37	0.696

*β*  standardized regression coefficient, *CI*  confidence interval

### Substance use as a moderator

Substance use (alcohol/e-cigarette use) was present in 1.5% of participants and did not significantly moderate the relationship between screen time/social media use and mental health outcomes. The only significant two interactions across the two analysis schemes were observed in Group 3 (Increasing-then-Decreasing Screen Time Use), where those with substance use had a 9.29-point higher depression t-score (95% CI: 5.38, 13.21) compared to non-users in Group 1, as well as in Group 3 (Sharp Increase to High Social Media Use), where those with unknown substance use had a 9.84-point higher depression t-score (95% CI: 2.08, 17.61) compared to those with non-use in Group 1 (Tables 3 and 4).

## Discussion

The current study examined the association between longitudinal screen time trajectories and subsequent anxiety and depression and explored whether alcohol and electronic cigarette use moderated these associations. Our findings confirm that greater screen time was associated with higher anxiety and depression symptoms in a demographically diverse cohort of adolescents from ages 9–10 to ages 12–13. These results align with research linking higher screen exposure to mental health concerns, including decreased self-esteem, increased stress, and poorer emotional regulation [10, 12]. Although higher screen use was associated with anxiety and depression, these effects were small and should be interpreted cautiously. Given mixed evidence in the broader literature, findings underscore the need for population health initiatives aimed at guiding healthy digital use, while avoiding overstating causal conclusions.

The most concerning trajectory was the increasing screen time group, which reached nearly 100 h per week. This group exhibited the highest levels of anxiety and depression, reinforcing concerns that prolonged digital exposure may exacerbate mental health risks [15]. Potential mechanisms include reduced physical activity, disrupted sleep, and passive engagement with digital content, all of which have been linked to increased stress, anxiety, and depression [22].

Social media use had a more nuanced relationship with mental health. While higher social media use was strongly associated with depression, it was not significantly associated with anxiety. These findings are consistent with studies indicating that social media may contribute to negative self-perception, cyberbullying, and comparison-based stress, all of which are associated with higher depression risk [11, 41].

Moderate social media use was associated with lower anxiety compared with low use. This may reflect that controlled social media engagement may provide social support, entertainment, or positive reinforcement that helps mitigate anxiety symptoms [42, 43]. However, high and fluctuating social media use correlated with significantly higher depression, highlighting the need for balance in digital engagement among adolescents. Taken together, these patterns suggest that general screen exposure relates broadly to both anxiety and depression, whereas social media use appears more specifically associated with depression. This distinction underscores the importance of differentiating digital activities in public health research and tailoring intervention strategies, such as general screen time management to address both anxiety and depression, and targeted approaches to mitigate the depressive symptoms associated with excessive social media use.

Despite expectations that substance use would modify the association between screen time and mental health outcomes, findings did not support this hypothesis. Two significant interactions emerged for high-increasing social media use, where unknown substance use correlated with higher depression t-scores, and an interaction between increasing-then-decreasing screen time use and substance use with higher depression t-scores. Since the moderation analysis was based on small numbers, findings should be interpreted with caution. This likely reflects the low prevalence of alcohol and electronic cigarette use (1.5%) in the sample, as most participants were 12–13 years old and had not yet engaged in significant substance use. Substance use initiation typically increases in later adolescence, so the full effect of substance use as a moderator may not be measurable in this younger cohort [4, 25]. Additionally, as increases in substance use are known to occur simultaneously with anxiety/depression in adolescence, the bidirectional nature of this association needs to be further examined in relation to screen time with an approach such as random intercept cross-lagged panel models [24]. Future research might also examine whether anxiety/depression acts as a potential moderator between screen time and substance use behavior. The key contribution of this study is that it identifies distinct patterns of screen and social media use among adolescents, showing how these trajectories relate to mental health outcomes.

Children and early adolescents engage in screen time behaviors that can be separated into three categories: entertainment (includes watching videos and playing video games), learning activities, and socializing via social media [6]. Our overall analysis showcases that increased time spent on screens may be associated with higher anxiety and depression, however, we were unable to determine whether a specific type of screen time use accounted for most of this effect. Previous research has highlighted the role of increased screen time in the form of social media use with poor health outcomes and increases in both anxiety and depression among youth [41, 44, 45]. Our results from the social media exploratory analysis indicate that in this population social media could be the driving force behind higher depression and not higher anxiety seen in our overall screen time analysis.

It is important to consider other potential factors that may play a role in the association between screen time use and mental health outcomes. Although both male and female adolescents have reported increased screen time use over time, sex differences regarding what modalities this screen time is allocated to have been previously described in the literature. While males spend more screen time playing computer and video games, females spend more time on social media and chatting online [46, 47] Additionally, it is well documented that depression and generalized anxiety are more prevalent among adolescent females compared to males, which represents an existing major health disparity [48]. Despite the fact that clear differences in adolescent screen time behavior and anxiety/depression have been documented between the sexes, the interaction of sex with the association between screen time and anxiety/depression remains inconclusive, which aligns with the results found in our study [49, 50]. Moreover, the literature has identified potential associations between socioeconomic factors and mental health outcomes. Adolescents who have faced higher stressors (such as low household income and low parental education) generally have a higher risk of developing depression and anxiety and reported higher screen time, compared to the youth with higher SES [51, 52]. However, the opposite pattern was found in this study, where White participants, and participants with parents with higher education showed higher risk of anxiety and depression scores compared to their counterparts. This pattern may reflect several interrelated mechanisms associated with higher parental education. Adolescents from more educated families may experience greater academic pressure and performance expectations, as well as increased awareness and reporting of symptoms, mirroring recent U.S. trends showing higher anxiety and depression among young people from higher-income households [53]. In addition, achievement-oriented or more controlling parenting styles may heighten stress sensitivity and limit emotional autonomy, contributing to internalizing symptoms [54]. Adolescents raised in enriched environments may also develop greater cognitive maturity and self-awareness, which can increase tendencies toward rumination and self-critical evaluation, further elevating risk for anxiety and depression [55]. This unexpected pattern warrants further study. Interventions may need to address underlying social determinants of health alongside digital behaviors.

Some strengths of the data include its demographically diverse nature, and that data was collected longitudinally for 9- to 13-year-olds. There was also a relatively large sample size of adolescents to analyze in the sample. Further, our use of GBTM allowed for data-driven classification of individuals into screen time and social media latent groups of homogenous individuals, which promoted objective groupings into varying trajectories where there is little within-class variation within individuals belonging to each trajectory group and introduced less selection bias than investigator led categorization in our analyses [40, 56]. Some limitations were that data are observational, so we were unable to detect causal relationships, and it is always possible that there are unmeasured confounders. In addition, screen time and social media use were relatively crude and reported by adolescents themselves without parental input and did not distinguish between different purposes (e.g., gaming, learning, socializing), which may differentially relate to mental health outcomes. As such, responses may be limited by recall and/or social desirability bias, which may lead to under- or overestimation over time. Because there is at least equal validity of child vs. parent reports of internalizing symptoms, we expect similar validity for child vs. parent screen time/social media reports [57]. In addition, the coding scheme for time spent on social media changed between the year 1 and year 2 follow-ups, although there did not appear to be a difference in comparing between the first and second half of the trajectory period for the Persistent Low Use group. Anxiety and depression t-score samples were right-skewed in the sample; however, the large sample allowed for parametric tests to be utilized. We were unable to assess for clinical significance of higher anxiety and depression t-scores as the CBCL is not validated for clinical assessment on its own. Further, screen and social media time were self-reported by adolescents, whereas anxiety and depression were parent-reported, which may introduce recall or information bias. Lastly, we did not control for potential third variables such as adverse childhood experiences, family stress, or school environment, which may confound associations. Future studies should incorporate these covariates.

## Conclusions

In conclusion, this study highlights the association between screen time and depression/anxiety among US adolescents. We found that screen time use increased over the three-year assessment period, and, importantly, that there was heterogeneity in adolescent screen time over time, with 4–5 different trajectory groups identified. While social media use was not associated with anxiety, it was associated with depression, especially in high-use groups. Alcohol/e-cigarette use did not significantly moderate these relationships, though its effects may emerge in later adolescence [58, 59]. Our findings underscore the potential importance of monitoring screen time and understanding the implications of screen time and mental health outcomes during adolescence. Parents may be encouraged to discuss digital behaviors with their children and set boundaries on excessive usage. Schools and communities may promote healthy screen habits, emphasizing time limits, digital detox strategies, and active over passive engagement for digital-well-being. Mental health programs could focus on helping adolescents navigate social media pressures and develop emotional resilience.

Targeting screen time management to improve mental health outcomes could be tailored to the identified groups. Additional considerations should be investigated in the future, including sensitivity analyses to determine whether sex plays an important moderating role, further analyses examining the bidirectionality of substance use behavior and anxiety/depression symptomology, and examining other forms of substance use and heavier use patterns. Furthermore, other factors, such as genetic factors, risk propensity, neural mechanisms, and temporal-behavioral patterns (e.g., schedules), could potentially moderate the association between screen time and mental health, and should be considered in future studies. Understanding the mechanisms connecting screen time with mental health is critical to target specific interventions and policies, such as limiting time on social media, that could contribute to the mental health of adolescents. At a public health level, monitoring screen time trajectories in early adolescence may help identify groups at higher risk for anxiety and depression. Prevention efforts should balance the potential risks of high use with opportunities for positive engagement, ensuring that digital health strategies are integrated into adolescent mental health promotion.

## Supplementary Information

Supplementary Material 1.

## Data Availability

Data from the Adolescent Brain Cognitive Development (ABCD) Study are available upon request from the NIH NIMH Data Archive (https://nda.nih.gov/abcd).

## Abbreviations

ABCD: Adolescent Brain and Cognitive Development
CBCL: Child Behavior Checklist
GBTM: Group-based trajectory modeling
