# Supplementary material for: Adolescent screen time, anxiety/depression, and alcohol/e-cigarette use: evidence from the ABCD study
Source: BMC Public Health. 2025 Dec 17;26:262. doi: 10.1186/s12889-025-25956-3 (PMC12822157; doi:10.1186/s12889-025-25956-3)
Supplement: Supplementary file 1 — Supplementary Material 1. [file 12889_2025_25956_MOESM1_ESM.docx]

**Supplementary Table 1.** Model diagnostics for intercept order trajectory group models stratified by screen time/social media, n = 9,474.

| **Screen Time** | | | | |
| --- | --- | --- | --- | --- |
| **Number of Groups** | **BIC** | **Entropy** | **Posterior Probability (for each group)** | **Group size (% of total n for each group)** |
| 2 | -165779.26 | 0.853 | 0.970/0.904 | 80.06/19.94 |
| 3 | -164938.76 | 0.810 | 0.939/0.844/0.890 | 68.43/26.36/5.21 |
| 4 | -164747.92 | 0.828 | 0.931/0.829/0.882/0.900 | 63.73/27.13/5.34/3.80 |
| 5 | -164799.35 | 0.834 | 0.864/0.926/0.805/0.853/0.879 | 0.03/65.11/26.89/7.08/0.89 |
| 6 | -164808.51 | 0.852 | 0.983/0.999/0.927/0.805/0.853/0.879 | 0.00/0.00/65.11/26.92/7.08/0.89 |
| **Social Media** | | | | |
| **Number of Groups** | **BIC** | **Entropy** | **Posterior Probability (for each group)** | **Group size (% of total n for each group)** |
| 2 | -67413.72 | 0.544 | 0.863/0.862 | 56.35/43.65 |
| 3 | -67145.78 | 0.641 | 0.844/0.837/0.794 | 30.44/63.66/5.90 |
| 4 | -67154.93 | 0.625 | 0.840/0.795/0.947/0.791 | 33.12/56.23/3.31/7.35 |
| 5 | -67138.66 | 0.612 | 0.879/.892/0.832/0.846/0.863 | 44.91/41.90/7.16/2.58/3.44 |
| 6 | -67167.83 | 0.532 | 0.717/0.747/0.448/0.860/0.687/0.747 | 32.52/0.36/29.51/29.42/8.06/0.12 |
